# Supplementary figures and images for: Changes in the gut microbiota of Nigerian infants within the first year of life
Source: PLoS One. 2022 Mar 17;17(3):e0265123. doi: 10.1371/journal.pone.0265123 (PMC8929609; doi:10.1371/journal.pone.0265123)

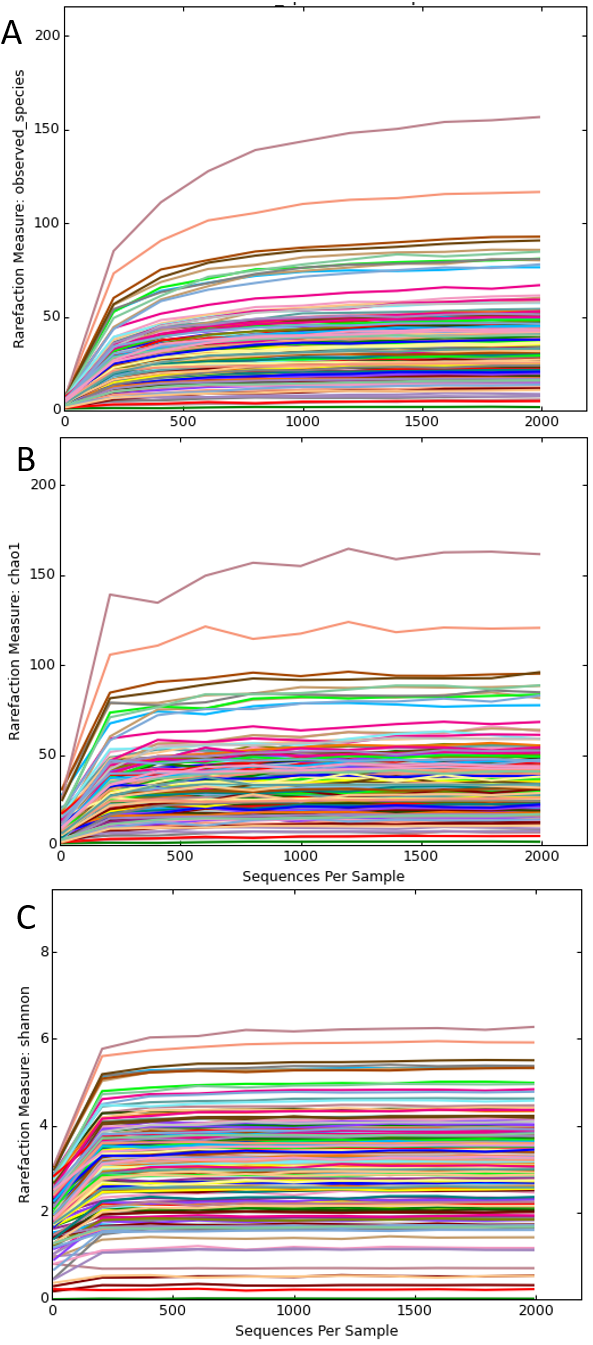

Supplement: S1 Fig — Analysis was performed using the core diversity analyses.py script from QIIME (version 1.9.0). (TIF) [file pone.0265123.s001.tif]
