# Supplementary material for: Changes in the gut microbiota of Nigerian infants within the first year of life
Source: PLoS One. 2022 Mar 17;17(3):e0265123. doi: 10.1371/journal.pone.0265123 (PMC8929609; doi:10.1371/journal.pone.0265123)
Supplement: S2 Fig — (DOCX) [file pone.0265123.s002.docx]

A)


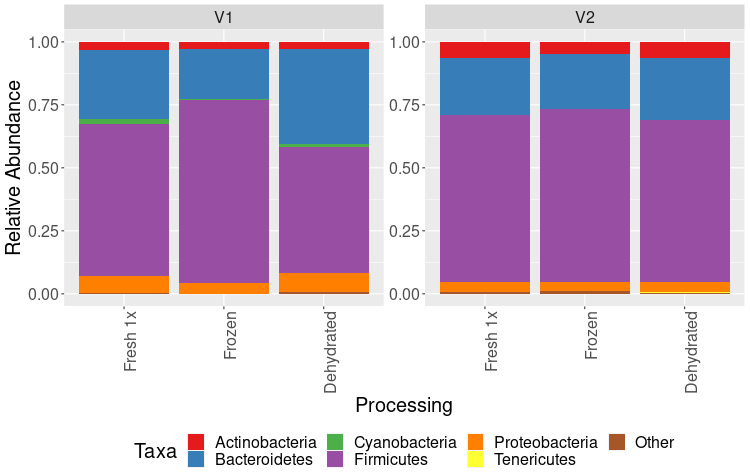


B)
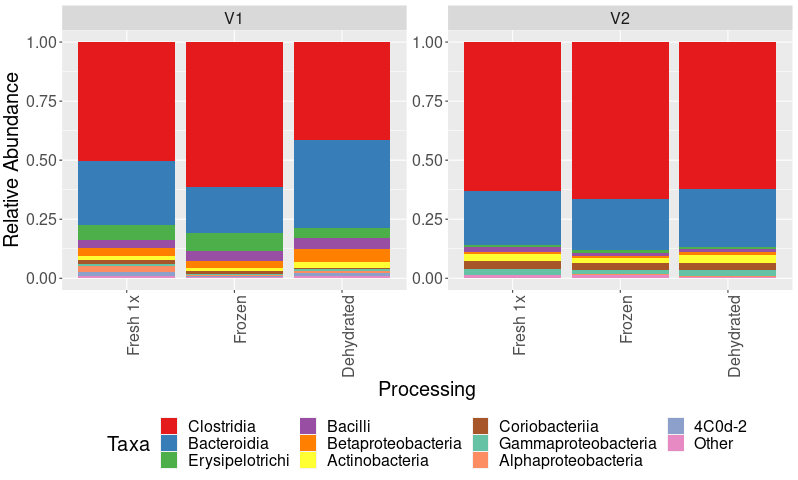


**S2 Fig. Bacterial composition profiles of samples from two volunteers to validate the desiccation storage method**. Samples from two volunteers (V1 and V2) were divided and processed fresh (fresh 1X), or stored for 3 months either frozen at -70°C (frozen) or desiccated (dehydrated) before DNA extraction and analysis by 16S rRNA gene sequencing following the outlined method. Subsequent microbial composition in samples after each of the three processing methods were compared at A) the phylum level or B) the class level.
