# Supplementary material for: Changes in the gut microbiota of Nigerian infants within the first year of life
Source: PLoS One. 2022 Mar 17;17(3):e0265123. doi: 10.1371/journal.pone.0265123 (PMC8929609; doi:10.1371/journal.pone.0265123)
Supplement: S3 Fig — (DOCX) [file pone.0265123.s003.docx]

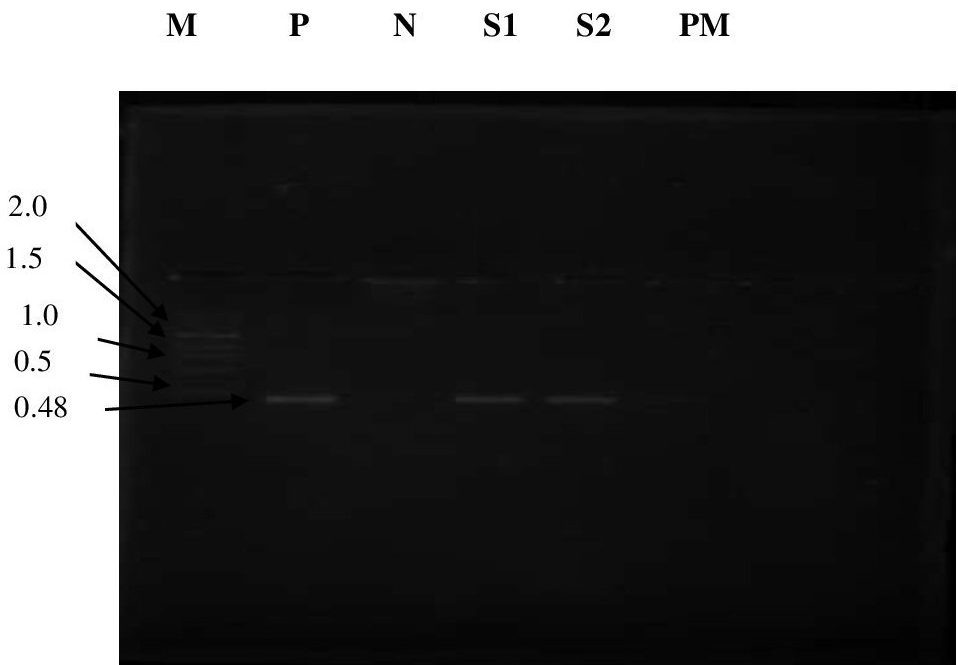


**S3 Fig. The presence of *Bifidobacterium* in NAN 1 formula milk.** Bifidobacteria were detected in formula milk consumed by the babies following PCR amplification using the generic *Bifidobacterium* set, Bif164f and Bif662r. The expected band of 480bp is clearly visible in the positive control (P) and two replicate extractions from the NAN 1 formula milk (S1 and S2). No bands are visible in the water-only negative control (N) nor in DNA extracted from the adult powered milk (PM). The sizes of bands in the marker ladder (M) are indicated in kbp.
